# *Toxocara* Seropositivity, Atopy and Wheezing in Children Living in Poor Neighbourhoods in Urban Latin American

**DOI:** 10.1371/journal.pntd.0001886

**Published:** 2012-11-01

**Authors:** Lívia Ribeiro Mendonça, Rafael Valente Veiga, Vitor Camilo Cavalcante Dattoli, Camila Alexandrina Figueiredo, Rosemeire Fiaccone, Jackson Santos, Álvaro Augusto Cruz, Laura Cunha Rodrigues, Philip John Cooper, Lain Carlos Pontes-de-Carvalho, Maurício Lima Barreto, Neuza Maria Alcantara-Neves

**Affiliations:** 1 Instituto de Ciências da Saúde, Universidade Federal da Bahia, Salvador, Bahia, Brazil; 2 Instituto de Matemática, Universidade Federal da Bahia, Salvador, Bahia, Brazil; 3 Instituto de Saúde Coletiva, Universidade Federal da Bahia, Salvador, Bahia, Brazil; 4 ProAR – Núcleo de Excelência em Asma, Universidade Federal da Bahia, Salvador, Bahia, Brazil; 5 London School of Hygiene and Tropical Medicine, University of London, London, United Kingdom; 6 Molecular and Biochemical Parasitology, Liverpool School of Tropical Medicine, Liverpool, United Kingdom; 7 Centro de Pesquisas Gonçalo Moniz, Fundação Oswaldo Cruz, Salvador, Bahia, Brazil; London School of Hygiene & Tropical Medicine, United Kingdom

## Abstract

**Background:**

*Toxocara canis* and *T. cati* are parasites of dogs and cats, respectively, that infect humans and cause human toxocariasis. Infection may cause asthma-like symptoms but is often asymptomatic and is associated with a marked eosinophilia. Previous epidemiological studies indicate that *T. canis* infection may be associated with the development of atopy and asthma.

**Objectives:**

To investigate possible associations between *Toxocara* spp. seropositivity and atopy and childhood wheezing in a population of children living in non-affluent areas of a large Latin American city.

**Methods:**

The study was conducted in the city of Salvador, Brazil. Data on wheezing symptoms were collected by questionnaire, and atopy was measured by the presence of aeroallergen-specific IgE (sIgE). Skin prick test (SPT), total IgE and peripheral eosinophilia were measured. *Toxocara* seropositivity was determined by the presence of anti-*Toxocara* IgG antibodies, and intestinal helminth infections were determined by stool microscopy.

**Findings:**

Children aged 4 to 11 years were studied, of whom 47% were seropositive for anti-*Toxocara* IgG; eosinophilia >4% occurred in 74.2% and >10% in 25.4%; 59.6% had elevated levels of total IgE; 36.8% had sIgE≥0.70 kU/L and 30.4% had SPT for at least one aeroallergen; 22.4% had current wheezing symptoms. Anti-*Toxocara* IgG was positively associated with elevated eosinophils counts, total IgE and the presence of specific IgE to aeroallergens but was inversely associated with skin prick test reactivity.

**Conclusion:**

The prevalence of *Toxocara* seropositivity was high in the studied population of children living in conditions of poverty in urban Brazil. *Toxocara* infection, although associated with total IgE, sIgE and eosinophilia, may prevent the development of skin hypersensitivity to aeroallergens, possibly through increased polyclonal IgE and the induction of a modified Th2 immune reaction.

## Author Summary

*Toxocara canis* and *T. cati* are roundworms found in dogs and cats, respectively, that can also infect humans and cause several clinical features, including asthma-like symptoms. Human infections with *T. canis* have been associated with an increased prevalence of atopy and asthma. In the present study, we investigated the associations between *Toxocara* seropositivity with eosinophilia, total IgE, specific IgE and skin prick test reactivity to aeroallergens, as well as atopic and non-atopic wheezing. *Toxocara* seropositivity was associated with elevated eosinophil counts and total and aeroallergen-specific IgE but was also associated with a decreased prevalence of skin prick test. *Toxocara* seropositivity was not associated with atopic wheezing. In conclusion, our data show that human toxocariasis, although associated with eosinophilia and raised levels of total and allergen-specific IgE, may play a role in the modulation of allergic effector responses in the skin.

## Introduction

There is evidence that the prevalence of allergic diseases has increased worldwide in recent decades, especially among populations living in large cities and living a Western lifestyle [1]. A better understanding of the causes and risk factors associated with this epidemic is important to identify novel preventive strategies against these diseases [2]. Epidemiological studies conducted in various geographic locations have shown that helminth infections are, under different circumstances, associated with a reduced or increased prevalence of atopy and allergic diseases [3], [4], [5], [6].

*Toxocara canis* and *T. cati* are intestinal roundworms found in dogs and cats, respectively, which may infect humans when exposed to their eggs in the environment. Humans serve as paratenic hosts in whom the parasites are unable to develop beyond the larval stage. Migratory *Toxocara* larvae may cause diseases in the liver, eyes, brain and lungs. Pulmonary toxocariasis has been reported to be associated with asthma-like symptoms [7]. Although several helminth infections of humans, such as *Trichuris trichiura* [8], [9], *Schistosoma mansoni* [10], [11], and *Ascaris lumbricoides* [12], [13], have been associated with a reduced prevalence of allergen skin test reactivity and asthma, human infection by *Toxocara* spp. has been associated with an increase in the prevalence of atopy and asthma symptoms [14]. Chan and collaborators [15] have previously shown that toxocariasis may increase predisposition to the development of allergic diseases, especially in children. It has also been demonstrated that toxocariasis is associated with elevated levels of specific IgE against aeroallergens (sIgE), serum total IgE, eosinophil counts [16], increased skin sensitivity to aeroallergens [17], atopic asthma in children [18], [19] and decreased lung function [20]. However, not all data support these associations: Zacharasiewicz and collaborators [21] were unable to show an association between *Toxocara* spp. seropositivity and allergen skin test reactivity, and they and others [18], [21], [22] did not observe an association between *Toxocara* infection and asthma.

The diagnosis of human toxocariasis is problematic because obtaining the excretory-secretory products of *Toxocara* larvae required for serologic assays is highly labour-intensive and time-consuming. Most serologic studies of human and animal toxocariasis use the excretory-secretory antigens of *T. canis* larvae (TcESLA) because *T. canis* females are easier to obtain from puppies. Due to the considerable antigenic cross-reactivity between the *Toxocara* larvae of both species, the detection of antibodies using the *T. canis* antigen does not discriminate between the two infections [23].

Because of the conflicting findings in the literature on the effects of *Toxocara* infection on atopy and asthma, we investigated this association in children living in poor urban neighbourhoods in Latin America where there is a high seroprevalence of specific IgG to *Toxocara* spp [24]. This study was carried out in the context of other chronic helminth infections of childhood that have also been associated with atopy and asthma [6]. After controlling for potential confounding factors, including intestinal helminths, we found that children who were seropositive for anti-*Toxocara* IgG had more eosinophilia and elevated levels of total and allergen specific IgE, which is consistent with the findings of previous studies [16], [17], [18], [19]. However, we also reported, for the first time in the literature, that *Toxocara* seropositivity was associated with a reduced prevalence of skin prick test (SPT) reactivity to common aeroallergens and that it may play an important role as an effect modifier in the association between sIgE and SPT.

## Methods

### Study population

This study was performed in the city of Salvador in Northeast Brazil, which has a population of 2,800,000. The study was performed with a cohort of 1,445 children aged 4 to 11 years who lived in non-affluent neighbourhoods and were chosen to represent areas of the city without sanitation. The cohort was chosen as part of a study conducted between 1997 and 2001 to assess the impact of a sanitation program on the occurrence of diarrhoea [25]. The children were resurveyed in 2005 to collect data on risk factors for wheezing [26]. The legal guardian of each child filled out an ISAAC Phase II-based questionnaire. Other social, demographic and environmental data were collected using validated questionnaires. Informed consent was obtained from the parents or guardians of the children, and ethical approval was granted by the Instituto de Saúde Coletiva da Universidade Federal da Bahia and the National Commission on Ethics in Research (CONEP), Brazil.

### Definitions of atopy and wheezing

Because the prevalence of sIgE for each of the studied allergens was greater than the SPT and the frequencies of SPT positivity among those without sIgE was very low [fungi (0.5%) dog epithelium (1.1%) and cat epithelium (0.9%)], atopy was defined as the presence of at least one positive test of the serum for anti-aeroallergen IgE≥0.70 kU/L (anti- *Dermatophagoides pteronyssinus*, *Blomia tropicalis*, *Blattella germanica* and *Periplaneta americana*), irrespective of SPT results.

Children were classified as currently wheezing if parents reported wheezing in the previous 12 months and the children had at least one of the following: (i) diagnosis of asthma ever, (ii) wheezing with exercise in the last 12 months, (iii) ≥4 episodes of wheezing in the last 12 months or (iv) waking up at night because of wheezing in the last 12 months. These questions were included to increase the specificity for current wheezing as a marker for asthma disease. All other children were classified as non-wheeziers. Atopic and non-atopic wheezings were defined as symptoms of wheezing in the presence or absence, respectively, of serum IgE ≥0.70 kU/L for any of the tested aeroallergens.

### Parasitological analysis

Two stool samples were collected from each child two days apart and analysed using the gravitational sedimentation [27] and Kato-Katz techniques [28] to detect eggs of *Ascaris lumbricoides*, *Trichuris trichiura*, hookworms and *Schistosoma mansoni*. Because hookworms and *Enterobius vermicularis* eggs were rarely observed (0.2% and 1.4%, respectively), these infections were excluded from the analyses. No *S. mansoni* eggs were observed in the stool samples.

### Collection of blood and skin prick test (SPT) exams

The children were evaluated in a mobile clinic in each of the study neighbourhoods, where they were evaluated by a medical team (doctor, nurse and laboratory technician), blood was collected (into EDTA-treated tubes), and skin prick testing for seven relevant aeroallergens was performed. At this time, the results of the stool examinations were provided to the parents, and appropriate treatment for parasite infections was given. Blood was taken to obtain differential blood cell counts (using an automated counter; Counter Electronics, Hialeah, FL, USA) and to measure total IgE, allergen-specific IgE and IgG to *Toxocara* spp. in plasma.

SPTs were performed on the right forearm of each child using extracts (ALK-Abello, São Paulo, Brazil) of *Dermatophagoides pteronyssinus*, *Blomia tropicalis*, *Blatella germanica*, *Periplaneta americana*, fungi, and cat and dog dander. Saline and 10 mg/mL histamine solution were used as negative and positive controls, respectively. Reactions were read after 15 minutes, and a mean wheal size of at least 3 mm greater than the negative control was considered positive.

### Measurement of total IgE and specific IgE to aeroallergens and to *A. lumbricoides*

The measurement of total IgE was performed as described previously [29]. Briefly, high binding microassay plates (Costar, Cambridge, ME, USA) were coated with 4 µg/mL of an anti-human IgE antibody (Pharmingen, San Diego, CA, USA) overnight at 4°C. Plates were blocked overnight at 4°C with PBS containing 10% foetal bovine serum (FBS) and 0.05% Tween 20. Samples were diluted 1∶10 in PBS containing 2.5% FBS and 0.05% Tween 20 and incubated overnight at 4°C. Plates were incubated sequentially with biotinylated anti-human IgE (Sigma Aldrich, San Louis, MO, USA), streptavidin/peroxidase (Pharmigen, San Jose, CA, USA) and substrate (a mixture of hydrogen peroxide and o-phenylenediamine; Sigma Aldrich, St Louis, MO, USA). Between all steps, the plates were washed three times with PBS containing 0.05% Tween 20 (PBS-T) and once with PBS. All incubations were for one hour at room temperature, except for the streptavidin-peroxidase and substrate steps, which were 30 minutes. A pool of sera from parasite-infected subjects was used as the positive control. Umbilical cord serum from a newborn of a non-atopic and non-parasitised mother was used as the negative control. The cut-off for elevated levels of total IgE was defined as 0.2 µg/ml, which represented the median plus the half the interquartile range for 54 negative control sera (from children with 3 consecutive stool samples that were negative for parasites, allergen-specific IgE levels of <0.35 kU/L, and <2% peripheral blood eosinophilia) [29].

Measurement of the levels of specific IgE to *B. tropicalis*, *D. pteronyssinus*, *P. americana*, *B. germanica* and *A. lumbricoides* was performed using the ImmunoCAP assay (Phadia Diagnostics AB, Uppsala, Sweden). These four specific mite and cockroach allergens were chosen to measure atopy based on the findings of skin prick test against a panel of seven relevant aeroallergens, which showed these to be the most relevant allergens in our study population. We used two cut-off points for aeroallergen-specific concentrations (≥0.35 kU/L and ≥0.70 kU/L) to investigate their association with *Toxocara* seropositivity; however, only the higher cut-off point was used to define atopy.

### Excretory/secretory products of *T. canis* larvae

Excretory/secretory products of *T. canis* larvae (TcESLA) were obtained as described previously [30] with appropriate modifications [31]. Briefly, puppies from parasite-infected bitches were treated with piperazine (100 mg/kg) and mineral oil. The uteri of adult *T. canis* females were dissected, the eggs removed and incubated in 2% formalin until embryonation. The egg membranes were disrupted using glass beads, and the released larvae were purified using a 15-µm pore polystyrene membrane filter. The larvae were cultured in RPMI medium (Sigma Chemical Co., St. Louis, USA) at 37°C in a CO_2_ incubator, and the culture supernatants containing TcESLA were cryopreserved at 70°C in the presence of 1 mM phenylmethylsulfonyl fluoride (PMSF; Sigma Chemical Co., St. Louis, USA) until use. TcESLA was concentrated using Amicon filters (Millipore Corporate, Billerica, MO, USA) with pores permeable to molecules of 3000 kDa and subsequently dialysed against phosphate buffered saline (PBS), pH 7.4. The protein content of the samples was determined using the Lowry technique (1951)[32], and the antigen was aliquoted and stored at 70°C, until further use.

### Absorption of sera with *A. lumbricoides* and *T. trichiura* extracts

To eliminate cross-reactive antigens shared by the ascarid worms *A. lumbricoides* and *Toxocara spp.*, human sera were absorbed with somatic antigens from *A. lumbricoides* before the measurement of anti-*Toxocara* IgG. *A. lumbricoides* antigen was prepared from adult worms obtained from children infected and treated with albendazole and 5 mg bisacodyl (Dulcolax). The worms were washed in saline and crushed in an electric grinder (Bead-Beart, Biospec, USA) in the presence of PBS that contained protease inhibitors [1 mM phenylmethylsulfonyl fluoride (PMSF), 2 mM ethylenediamine tetra-acetic acid (EDTA), 2 mM tosyl phenylalanyl chloromethyl ketone (TPCK), and 50 µM p-tosyl-L-lysine chloromethyl ketone (TLCK) (Sigma Chemical Co., St. Louis, USA)]. The suspension was centrifuged, and the soluble fraction stored at −70°C after determining the protein concentration using the Lowry method [32]. For absorption of sera, 100 µL of each serum was incubated with 250 µL of a solution containing: 4.0 mg/mL *A. lumbricoides* antigen, 100 µL polyethylene glycol (Sigma Chemical Co., St. Louis, USA) and 50 µL PBS. After incubation for 30 minutes at room temperature under agitation, the material was centrifuged for 10 minutes. The supernatant was collected, re-absorbed with *A. lumbricoides* antigen and kept at −70°C until assayed. The second absorption was performed because some cross-reactive antibodies remained in the sera after the first absorption. Because 10.7% of the children were infected with *T. trichiura*, a sample of the studied sera was also absorbed with this parasite extract and compared to the same sera absorbed with *A. lumbricoides* alone or with both parasites. Because absorption with *A. lumbricoides* alone or with both parasites provided comparable titers of anti-*Toxocara* IgG, the remaining sera were absorbed with *A. lumbricoides* antigen alone.

### Detection of serum anti-*Toxocara* IgG antibodies

The detection of anti-*Toxocara* IgG antibodies was carried out as previously described by de Savigny with modifications [33]. Briefly, 96-well plate wells were incubated overnight at 4°C with 3.2 µg/mL of TcESLA in pH 9.6 carbonate/bicarbonate buffer. The plates were blocked with 0.15 M phosphate-buffered saline, pH 7.4 (PBS), containing 10% FBS (Sigma Aldrich, St Louis, MO, USA). Sera that had been pre-absorbed with *A. lumbricoides* extract and diluted at 1∶1000 in PBS containing 0.05% Tween 20 and 2.5% FBS (PBS-T-FBS) were added to the plates. After incubation, a solution of biotinylated anti-human IgG (BD, Pharmigen, San Jose, CA, USA) was added, followed by incubations with streptavidin-peroxidase (BD, Pharmigen, San Jose, CA, USA), and substrate (Sigma Aldrich, St Louis, MO, USA). Washings, incubations and reading were performed as described above for the measurement of total serum IgE. The reaction was blocked with 2 N sulphuric acid and read using a spectrophotometer at 490 nm (Biotek EL-800, CA, USA). The cut-off for the assay was obtained using the mean plus three standard deviations of the anti-*Toxocara* IgG assay from the negative control sera, which were obtained from 20 children without contact with dogs and cats. Because this assay does not discriminate infection by *T. canis* or *T. cati*, we used the results of this assay as marker of past or present infection by both *Toxocara* species [23].

### Statistical analysis

For the associations of *Toxocara* infection with eosinophilia, total IgE, aeroallergen-specific IgE, SPT and asthma, univariate and multivariate analyses were performed using logistic regression. Atopic and non-atopic wheezing were defined as current wheezing in the presence or absence, respectively, of ≥0.70 kU/L specific IgE to at least one aeroallergen. *A priori* confounders for the association between *Toxocara* seropositivity and outcomes were gender and age. The following potential confounders were considered: body mass index (BMI), maternal educational level, parental asthma, parental smoking, household connection to the municipal sewage system, living on a paved street, frequency of garbage collection, number of siblings, the presence of cat(s) and/or dog(s) in the house, the presence of mould or dampness on the walls of the house (by inspection), the presence of cockroach and rodents at home, attendance at day-care centre and period of attendance, and presence of *A. lumbricoides and T. trichiura* infections in stool samples. These variables were selected because they were associated with seropositivity to *Toxocara* or atopy or asthma in univariate analyses (Table 1) or because they had been identified as confounders in a previous analysis using data from these children [34]. To build multivariate logistic regression models, we used a procedure in which step-wise forward selection of variables was performed. Significant variables from the univariate analysis were included, and each non-significant variable was included sequentially; if a variable became significant, it was kept in the model, but if it remained non-significant, it was discarded. The interaction of *Toxocara* seropositivity with the association between SPT and sIgE was analysed by univariate regression analysis, and the statistical significance of the interaction was provided by the Breslow-Day's test for odds ratio homogeneity.

**Table 1:** Frequencies of the studied variables and their associations with anti-*Toxocara* IgG seroposivity in 1,148 children.

*Variables*	N	%	*Anti-Toxocara* IgG seropositivity n = 540 (47.0%)	
			n(%)	Crude OR (95% CI)
**Gender**				
Female	532	46.3	243(45.7)	1
Male	616	53.7	297(48.2)	1.11(0.88; 1.40)
**Age (years)**				
≤5	298	26.0	133(44.6)	1
6–7	465	40.5	203(43.7)	0.96(0.72; 1.29)
≥8	385	33.5	204(53.0)	**1.40(1.03; 1.89)**
**Maternal Schooling**				
1st grade or less	251	21.9	149(59.4)	**1**
Incomplete 2nd grade	554	48.3	280(50.5)	**0.70(0.52; 0.95)**
Complete 2nd grade or more	343	29.9	111(32.4)	**0.33(0.23; 0.46)**
**Parental Asthma**				
No	994	86.6	465(46.8)	1
Yes	154	13.4	75(48.7)	1.08(0.77; 1.52)
**Mold at household**				
No	345	30.1	167(48.4)	1
Yes	803	69.9	373(46.5)	0.92(0.72; 1.19)
**Connection to sewage system**				
No	194	16.9	109(56.2)	1
Yes	954	83.1	431(45.2)	**0.64(0.47; 0.88)**
***Ascaris and/or Trichuris***				
No	914	79.6	375(41.0)	1
Yes	234	20.4	165(70.5)	**3.44(2.52; 4.69)**
**Eosinophilia**				
<**4%**	296	25.8	80 (27)	1
≥**4%**	852	74.2	460 (54)	**3.17(2.35; 4.27)**
<**10%**	856	74.6	345 (40.3)	1
≥**10%**	292	25.4	195 (66.8)	**2.98(2.23; 3.97)**
**Total IgE**				
<0.2 µg/ml	464	40.4	186 (40.1)	1
≥0.2 µg/ml	684	59.6	354 (51.8)	**1.60(1.26; 2.04)**
* **Specific IgE reactivity**				
<0.35 kU/L	591	51.5	252 (42.6)	1
≥0.35 kU/L	557	48.5	288 (51.7)	**1.44(1.14; 1.82)**
<0.70 kU/L	726	63.2	327 (45)	**1**
≥0.70 kU/L	422	36.8	213 (50.5)	1.24(0.98; 1.58)
* **Skin prick test reactivity**				
No	797	69.4	394 (49.4)	**1**
Yes	351	30.6	146 (41.6)	**0.73(0.56; 0.94)**
** ***Wheeze plus asthma symptoms***				
No	890	77.5	409 (46)	1
Yes	258	22.5	131 (50.8)	1.21(0.92; 1.60)

* For at least one of the tested allergens.

** Wheeze plus (i) diagnosis of asthma ever; (ii) wheezing with exercise in the last 12 months; (iii) ≥4 episodes of wheezing in the last 12 months; (iv) waking up at night because of wheezing in the last 12 months. Boldface numbers show those that are statistically significant at p<0.05.

To analyse the association of *Toxocara* seropositivity with wheeze phenotypes (atopic x non-atopic), multivariate logistic regression analyses were performed as described previously [35]. Thus, non-atopic wheeziers were compared with non-atopic non-wheeziers (to estimate the risk of wheezing associated with toxocariasis among non-atopic children), while atopic wheeziers were compared separately with two groups to demonstrate the importance of choosing the appropriated reference group and the differences generated when different comparison groups are chosen: group 1 - non-atopic and non-wheeziers (to estimate risk of wheezing associated with toxocariasis among atopic children uncontrolled by the effect of atopy); and group 2 - atopic and non-wheeziers (to estimate risk of wheezing associated with toxocariasis among atopic children controlled by the effect of atopy). We used multinomial logistic regression because it treats the categories of the polytomy (atopic wheeziers, non-atopic wheeziers, atopic non-wheeziers, and non-atopic non wheeziers) in a non-arbitrary order and also addresses several sets of log-odds that correspond to different dichotomies.

## Results

Of the 1,445 children enrolled in the study, complete data were obtained from 1,148, all of which were included in the analysis. No statistically significant differences were observed in the prevalence of the outcomes when excluded children (n = 297) were compared with those included in the analysis (data not shown).

Table 1 shows the distribution of study variables and outcomes among the study children and the associations with *Toxocara* seropositivity, as analysed by univariate analysis. Seroprevalence of *Toxocara* IgG increased with age and was greater among children with *A. lumbricoides* and T. *trichiura* infections and among those without a household connection to the sewage system. *Toxocara* seropositivity was positively associated with eosinophilia and with high levels of total and specific IgE (≥0.35 and ≥0.70 kU/L) and was negatively associated with skin prick test (SPT) reactivity.

Tables 2 and 3 show the multivariate logistic analysis of the association between *Toxocara* seropositivity with the study outcomes. Models were adjusted for the following confounders: gender, age, maternal schooling, parental asthma, presence of mould, sewage access and infections with *A. lumbricoides* and *T. trichiura*. Positive associations were observed between the *Toxocara* seropositivity and total IgE and eosinophilia (at cut-offs of >4% and >10%) (Table 2). The presence of sIgE, defined using cut-offs of ≥0.35 and ≥0.70 kU/L for at least one tested allergen, was also positively and significantly associated with *Toxocara* seropositivity. A statistically significant inverse association was observed between *Toxocara* seropositivity and SPT (Table 3). When anti-*Toxocara* IgG was stratified by optical density (to represent levels of anti-*Toxocara* IgG), dose-response associations were observed, such that greater optical densities were associated with a greater prevalence of all study outcomes (Tables 2 and 3), with the exception of SPT, in which higher optical densities were associated with a reduced prevalence of SPT.

**Table 2:** Associations between anti-*Toxocara* IgG seropositivity and total IgE and eosinophilia of ≥4% and ≥10% in 1,148 children.

Anti-*Toxocara* IgG seropositivity	*Total IgE* *		*Eosinophilia (4%)*		*Eosinophilia (10%)*	
	n (%)	**OR (95% C.I.)	n (%)	**OR (95% C.I.)	n (%)	**OR (95% C.I.)
Negative (n = 608 53.0%)	330 (54.3)	1	392 (64.5)	1	97 (16.0)	1
Positive (n = 540 47.0%)	354 (65.6)	**1.53 (1.19; 1.97)**	460 (85.2)	**3.03 (2.22; 4.13)**	195 (36.1)	**2.46 (1.83;3.29)**

Anti-*Toxocara* IgG *l*evels***	n (%)	*OR (95% C.I.)	n (%)	*OR (95% C.I.)	n (%)	*OR (95% C.I.)
Neg <0.23 (n = 608 53.0%)	330 (54.3)	**1**	392 (64.5)	**1**	97 (16.0)	**1**
≥0.23 and <1.0 (n = 329 28.7%)	212(64.4)	**1.48 (1.12; 1.97)**	265 (80.5)	**2.35 (1.67; 3.29)**	82 (24.9)	**1.55 (1.10; 2.19)**
≥1.0 (n = 211 18.4%)	142(67.3)	**1.63 (1.15; 2.32)**	195 (92.4)	**5.94 (3.41; 10.36)**	113 (53.6)	**4.69 (3.23; 6.80)**

* Positivity for total IgE defined by a cut-off of 0.2 µg/mL;

** OR adjusted for gender, age, maternal schooling, parental asthma, mold, sewage access, infections with *A.lumbricoides* and *T. trichuris*.

*** Shown are strata of optical densities to represent antibody levels Boldface numbers show those that are statistically significant at P<0.05.

**Table 3:** Associations between anti-*Toxocara* IgG seropositivity and specific IgE (defined by (≥0.35 and ≥0.70 kU/L) and skin prick test (SPT) reactivity in 1,148 children.

Anti-*Toxocara*IgG seropisitivity	#IgE (≥0.35 kU/L)		#IgE (≥0.70 kU/L)		#SPT	
	n (%)	*OR (95%CI)	n (%)	*OR (95% CI)	n (%)	*OR (95%CI)
Negative (n = 608 53.0%)	269 (44.2)	1	209 (34.4)	1	205 (33.7)	1
Positive (n = 540 47.0%)	288 (53.3)	**1.51 (1.18; 1.94)**	213 (39.4)	**1.34 (1.03;1.73)**	146 (27.0)	**0.74 (0.57; 0.97)**

**Anti-*Toxocara* IgG *l*evels	n (%)	*OR (95%CI)	n (%)	*OR (95% CI)	n (%)	*OR (95%CI)
Negative <0.23 (n = 608; 53.0%)	269 (44.2)	1	209 (34.4)	1	205 (33.7)	1
≥0.23 to <1.0 (n = 329; 28.7%)	162 (49.2)	1.27 (0.96; 1.67)	122 (37.1)	1.17 (0.87; 1.57)	98 (29.8)	0.81 (0.60; 1.10)
≥1.0 (n = 211; 18.4%)	126 (59.7)	**2.08 (1.48; 2.94)**	91 (43.1)	**1.71 (1.20; 2.42)**	48 (22.7)	**0.61 (0.42; 0.90)**

# For at least one of the tested allergens;

* OR adjusted by gender, maternal schooling, parental asthma, mold, sewage system, infection by *A.lumbricoides* and *T. Trichuris*;

** Shown are strata of optical densities to represent antibody levels; Boldface numbers show those that are statistically significant at P<0.05.

The effect of *Toxocara* seropositivity on the association between sIgE and SPT positivity was analysed. We found that the association of sIgE with SPT increased with an increase in the sIgE levels and that this association was weaker in *Toxocara* seropositive children (Table 4).

**Table 4:** Effect of *Toxocara spp*. seropositivity in the association of sIgE with SPT reactivity in the 1,148 studied children.

*Toxocara* seronegative			*Toxocara* seropositive		*p-value
#sIgE (kU/L)	#SPT		#SPT		
	n(%)/N	Crude OR (95% CI)	n(%)/N	Crude OR (95% CI)	
Negative	36(9.0)/399	**1**	32(9.8)/327	**1**	
Positive	169(80.9)/209	**42.60 (26.21; 69.25)**	114(53.5)/213	**10.62 (6.75; 16.70)**	**<0,001**

#sIgE levels	n (%)	*OR (95%CI)	n (%)	*OR (95% CI)	n (%)
<0.70	36(9.0)/399	**1**	32(9.8)/327	**1**	
0.70 to <3.5	42(55.3)/76	**12.46 (6.58; 23.59)**	44(35.2)/125	**5.01 (2.91; 8.63)**	**0,0316**
≥3.5	127(95.5)/133	**213.43 (46.55; 978.58)**	70(79.5)/88	**35.85 (15.38; 83.59)**	**0.0239**

# For at least one tested allergen;

* Breslow-Day test for odds ratio homogeneity; Boldface numbers show those that are statistically significant at P<0.05.

We evaluated the associations between *Toxocara* seropositivity and the levels of anti-*Toxocara* IgG antibodies with wheezing phenotypes. The results are shown in Table 5. A positive and weak association (statistically non-significant, but borderline) was found between high levels of anti-*Toxocara* IgG and non-atopic asthma. Although a positive association between anti-*Toxocara* IgG seropositivity and atopic wheezing was found when non-atopic non-asthmatic children were used as reference group, this association disappeared when we used the appropriate reference group (atopic non-asthmatic children), as recommended previously by Barreto and colleagues [35].

**Table 5:** Polytomous logistic regression analysis comparing the associations between anti-*Toxocara*IgG seropositivity and non-atopic wheeze and atopic wheeze phenotypes in 1,148 children.

Anti- *Toxocara* IgG seropositivity (N = 1,148)	Non-atopic wheeze plus asthma symptoms#		Atopic wheeze plus asthma symptoms#			
	Reference group non-atopic, non-wheeziers		Reference group non-atopic, non-wheeziers		Reference group atopic non-wheeziers	
	N = 706		N = 706		N = 422	
	n (%)/N	** OR (95% CI)	n (%)/N	** OR (95% CI)	n (%)/N	**(95% CI)
Negative	69(17.3)/399	1	58(14.9)/388	1	58(27.8)/209	1
Positive	70(21.4)/327	1.19 (0.80; 1.77)	61(19.2)/318	**1.57 (1.03; 2.39)**	61(28.6)/213	1.16 (0.74; 1.82)

**Anti -*Toxocara* IgG *l*evels	n (%)/N	* OR (95% CI)	n (%)/N	* OR (95% CI)	n (%)/N	* (95% CI)
Negative<0.23	69(17.3)/399	1	58(14.9)/388	1	58(27.8)/209	1
≥0.23 and <1.0	35(16.9)/207	0.94 0.59; 1.50)	34(16.5)/206	1.29 (0.80; 2.10)	34(27.9)/122	1.15 (0.69; 1.94)
≥1.0	35(29.2)/120	1.63 (0.98; 2.75)	27(24.1)/112	**2.19 (1.25; 3.84)**	27(29.7)/91	1.20 (0.66; 2.17)

# Wheeze plus: (i) diagnosis of asthma ever; (ii) wheezing with exercise in the last 12 months; (iii) ≥4 episodes of wheezing in the last 12 months; (iv) waking up at night because of wheezing in the last 12 months.

* OR adjusted by gender, maternal schooling, parental asthma, mold, sewage system, infection by *A.lumbricoides* and *T. Trichuris*;

** Shown are strata of optical densities to represent antibody levels; Boldface numbers show those that are statistically significant at P<0.05.

## Discussion

Human infections with *Toxocara* spp. are generally difficult to diagnose because the parasites are inaccessible and most infections are asymptomatic. Thus, the prevalence of toxocariasis is grossly underestimated, posing a significant challenge to investigators interested in evaluating the public health impact of this infection [36]. Previous estimates of *Toxocara* seroprevalence in Latin America are highly variable, ranging from 4% to 52% in Brazil [37]. The latter prevalence was reported among adults in a village in the Amazon region [38]. Prevalences of 38% and 32% have been reported in children from Argentina [39] and Peru [40], respectively. A previous study in Salvador, where the present study was performed, estimated a prevalence of 46% among blood donors with eosinophilia but no evidence of intestinal helminth infection [24], which was similar to the high seroprevalence of 47% that was observed in the present study among children living in poor urban neighbourhoods and who were not selected by eosinophilia or helminth-infection status.

Our data show that *Toxocara* seroprevalence was associated with increasing age, low levels of maternal educational, a lack of household access to the municipal sewage system, and co-infections with intestinal helminths. Such factors are likely markers of poverty and poor hygiene, under which circumstances children are at greater risk of acquiring a number of infections [6], including from their pets or other neighbourhood animals, which are rarely, if ever, dewormed. *Toxocara* seropositivity was found associated with both cats and dogs at home in the studied population (submitted data). A previous analysis in the same studied children of eight taxonomically distinct pathogen exposures, including viral, bacterial, protozoal and helminth (*A. lumbricoides* and *T. trichiura*) infections, showed strong positive associations between multiple infections, indicating shared risk factors [6]. Risk factors related to poverty and poor hygiene are common in toxocariasis [41]. The association between *Toxocara* seropositivity and intestinal helminth infections could also be explained by immunological cross-reactivity, although we believe that any false positive serologic reactions associated with intestinal helminths would have been minimised by the extensive absorption of sera with *A. lumbricoides* antigens carried out before measurement of *Toxocara* antibodies, as well as by the high serum dilution used in this assay.

The present study investigated the relationship between *Toxocara* seropositivity and several markers of allergic-type responses and allergic disease, including eosinophilia, total IgE, markers of atopy, and atopic and non-atopic wheezing. Typically, helminth infections, such as by *Toxocara* spp., stimulate Th2 immune responses, a type of immune response that is considered to be central to the development of atopy and allergy. Experimental infections of mice with *T. canis* have been associated with increased inflammatory activity, intense migration of eosinophils to the lungs and increased plasma levels of pro-inflammatory cytokines such as IL-6 and IFN-γ and eosinophil-associated chemokines such as eotaxin and RANTES [42]. Our findings demonstrate that *Toxocara* seropositivity was associated with high levels of total IgE and eosinophilia, even after adjustment for co-infections with intestinal helminths, confirming that *Toxocara* infection is a strong inducer of IgE and eosinophilia. Previous studies have demonstrated that eosinophilia is present in up to 87% of individuals with toxocariasis [40], [43].

*Toxocara* seropositivity was also associated with the presence of specific IgE to mite and cockroach allergens. There is evidence from several studies of extensive cross-reactivity between mites and helminth parasites: 1) Johansson and collaborators (2001) [44] reported cross-reactivity of IgE antibodies between a fish nematode (*Anisakis simplex*) and mites (*Acarus siro*, *Lepidoglyphus destructor*, *Tyrophagus putrescentiae* and *D. pteronyssinus*), 2) Ponte and collaborators (2011) [45] reported a high frequency of cross-reactive IgE antibodies between *B. tropicalis* and *A. lumbricoides* (an ascarid worm that is closely related to *Toxocara* spp.), and 3) Acevedo and collaborators (2009) [46] reported the presence of multiple antigens that were cross-reactive between *A. lumbricoides* and *B. tropicalis*, including tropomyosin and glutathione-S-transferase. Despite using a more stringent cut-off for positivity for allergen-specific IgE (≥0.70 kU/L rather than that usually recommended for the definition of atopy, ≥0.35 kU/L) in the present study to minimise the problem of cross-reactivity of low-affinity IgE, we observed a positive association between sIgE and *Toxocara* seropositivity. This observation could be explained by cross-reactivity between arthropod allergens and *Toxocara* antigens as described above [45], [46] by inducing production of allergen-specific IgE by plasma cells through polyclonal signals associated with helminth infections such as IL-4 or by the fact that children who develop strong Th2 responses to *Toxocara* may be more ‘atopic’ in the sense that they are more likely to develop IgE responses to environmental allergens.

Although we observed a positive association between *Toxocara* seropositivity and the presence of sIgE, *Toxocara* seropositivity was inversely associated with SPT to the same aeroallergens, and it had a strong modulator effect on the association between sIgE and SPT. The absence of allergen-specific skin reactivity, despite high sIgE values in the same individuals, has several possible explanations, including the following: 1) ‘mast cell saturation’ - the presence of high levels of parasite-induced polyclonal IgE ‘saturates’ high-affinity FcεR1 receptors on mast cells, thus reducing the probability that allergen cross-linking of specific IgE will lead to cell activation [47]; 2) IgG blocking antibodies, particularly those of the IgG4 class, may bind allergen epitopes, thus preventing access of such epitopes to specific IgE antibodies bound to the mast cell [47]; 3) cross-reactive carbohydrate determinants - cross-reactive IgE antibodies, which are reactive to common carbohydrates shared by mites and helminths such as phosphorylcholine-modified glycans or glycans containing Galb1-4(Fuca1-3)GlcNAc- (Lewis X, LeX), have low affinity to the allergen epitopes, and weak binding may reduce the chance of cross-linking of the IgE bound to FcεR1 receptors on the mast cell surface [48] (this phenomenon has been described for plants and pollen allergens [49]); and 4) the downmodulation of SPT has been attributed to the so-called “modified Th2 response” [3], [50], [51], in which helminth infection induces regulatory populations of T cells that produce immune regulatory cytokines such as IL-10, which may increase the threshold for mast cell activation [52]. Therefore, the negative association between anti-*Toxocara* antibody seropositivity and SPT found in this work could be due to at least two different phenomena. First, the infection by *Toxocara* could modulate the immune system so that the ability of sIgE to mediate SPT is reduced (e.g., by competition with *T. canis*-elicited polyclonal/cross-reactive IgE). This phenomenon is consistent with the findings that the whole anti-*Toxocara* antibody seropositive sub-group had higher sIgE levels than the anti-*Toxocara* antibody seronegative sub-group (Table 3). Second, immunological phenomena, such as IL-10 production, that is induced by the *Toxocara* infection could break the association between sIgE and SPT. In this case, one would expect a weaker association between sIgE levels and SPT reactivity, as found in anti-*Toxocara* antibody seropositive children (Table 4). While *Toxocara* seropositivity was strongly associated with sIgE in our study population, it was not associated with atopic wheezing. In contrast, previous studies have observed associations between *T. canis* seropositivity and increased skin sensitivity to allergens [20] and atopic asthma [7]. However, such studies were performed in different populations from different geographic regions and included adults.

We found a weak positive and statistically non-significant but borderline association between *Toxocara* seropositivity and non-atopic wheezing among children with the highest levels of anti-*Toxocara* IgG. However, a limitation of our study was the lack of power to show a statistically significant association of this finding. This finding may be explained by lung infestations with *Toxocara* larvae, which are known to cause asthma-like symptoms. Further limitations of our study were the cross-sectional study design, which did not allow us to distinguish exposure (presumed to be *Toxocara* infections) from our study outcomes (allergic and atopic markers and wheezing). We identified *Toxocara* infection using the presence of specific IgG antibodies - the presence of antibodies does not distinguish present from past infections. Similarly, we cannot preclude confounding by other helminth infections. For example, we did not measure pinworm infections, which are universal and require specific detection methods. There is extensive cross-reactivity between different helminth infections, but we tried to reduce false-positive reactions by pre-absorption of sera with *A. lumbricoides* antigens and using sera with the highest dilution described in the literature. This step was an important strength of our study. Other strengths were the use of a large sample of children, two markers for atopy (sIgE and SPT) and distinct control groups that allowed us to distinguish more clearly the effects of *Toxocara* seropositivity on atopy and wheezing.

The apparent protective effects of chronic helminth infections against atopy and the clinical manifestation of allergy and autoimmune diseases are counterbalanced by the adverse effects of these infections on childhood growth and nutrition and possible adverse effects on the immune response to vaccines [53]. Given that the prevalence of intestinal helminths and schistosomiasis has declined dramatically in Latin American countries such as Brazil over recent years, *Toxocara* infection is now likely to assume greater importance as a neglected public health problem and the most common endemic helminth infection in these countries.

The data from the present study revealed that almost half the children aged between 4 and 11 years living in poor neighbourhoods in a large city in the tropical region of Brazil have evidence of infection with *Toxocara*. Although this infection was associated with enhanced inflammatory markers (eosinophilia, total IgE) and an increased prevalence of sIgE, it appeared to be protective against immediate hypersensitivity reactions in the skin induced by common aeroallergens.
